# Cardioprotective Effects of Tualang Honey: Amelioration of Cholesterol and Cardiac Enzymes Levels

**DOI:** 10.1155/2015/286051

**Published:** 2015-05-03

**Authors:** Md. Ibrahim Khalil, E. M. Tanvir, Rizwana Afroz, Siti Amrah Sulaiman, Siew Hua Gan

**Affiliations:** ^1^Laboratory of Preventive and Integrative Biomedicine, Department of Biochemistry and Molecular Biology, Jahangirnagar University, Savar, Dhaka 1342, Bangladesh; ^2^Department of Pharmacology, School of Medical Sciences, Universiti Sains Malaysia, 16150 Kubang Kerian, Kelantan, Malaysia; ^3^Human Genome Centre, School of Medical Sciences, Universiti Sains Malaysia, 16150 Kubang Kerian, Kelantan, Malaysia

## Abstract

The present study was designed to investigate the cardioprotective effects of Malaysian Tualang honey against isoproterenol- (ISO-) induced myocardial infarction (MI) in rats by investigating changes in the levels of cardiac marker enzymes, cardiac troponin I (cTnI), triglycerides (TG), total cholesterol (TC), lipid peroxidation (LPO) products, and antioxidant defense system combined with histopathological examination. Male albino Wistar rats (*n* = 40) were pretreated orally with Tualang honey (3 g/kg/day) for 45 days. Subcutaneous injection of ISO (85 mg/kg in saline) for two consecutive days caused a significant increase in serum cardiac marker enzymes (creatine kinase-MB (CK-MB), lactate dehydrogenase (LDH), and aspartate transaminase (AST)), cTnI, serum TC, and TG levels. In addition, ISO-induced myocardial injury was confirmed by a significant increase in heart lipid peroxidation (LPO) products (TBARS) and a significant decrease in antioxidant enzymes (SOD, GPx, GRx, and GST). Pretreatment of ischemic rats with Tualang honey conferred significant protective effects on all of the investigated biochemical parameters. The biochemical findings were further confirmed by histopathological examination in both Tualang-honey-pretreated and ISO-treated hearts. The present study demonstrates that Tualang honey confers cardioprotective effects on ISO-induced oxidative stress by contributing to endogenous antioxidant enzyme activity via inhibition of lipid peroxidation.

## 1. Introduction

Oxidative stress due to excess production of reactive oxygen species (ROS), along with free radicals, has been implicated in a large number of diseases, including cardiovascular diseases (CVDs), liver diseases, and neurodegenerative diseases including Alzheimer's and Parkinson's disease [1, 2]. Myocardial infarction (MI) is a presentation of ischemic heart disease (IHD) that is defined as an acute condition of necrosis of the myocardium resulting from an imbalance between myocardial metabolic demands and the coronary supply of oxygen and nutrients. IHD is one of the most lethal manifestations of cardiovascular disease [3–5]. According to the World Health Organization, MI is the major cause of death in the developed world and a cause of major pathology worldwide. By 2020, MI is predicted to be the major cause of death in the world [6, 7].

Oxidative deterioration of membrane polyunsaturated fatty acids (PUFAs) within the myocardium has been linked to increased levels of lipid peroxides such as thiobarbituric acid reactive substances (TBARS) and lipid hydroperoxides (LOOH). These changes occur during the initial stages of MI and increase the tissue's susceptibility to oxidative damage. This is followed by hyperglycemia, hyperlipidemia, peroxidation of membrane phospholipids, and loss of membrane integrity [4, 8].

Isoproterenol (ISO) is a synthetic catecholamine and *β*-adrenergic agonist. When administered in large doses, it can cause severe stress in the myocardium that leads to infarct-like necrosis of the heart muscle [9]. It has been reported that ISO-induced cardiac damage is characterized by hypoxia due to myocardial hyperactivity, coronary hypotension, and excessive generation of highly cytotoxic free radicals as a result of auto-oxidation of catecholamines [10]. The oxidation of catecholamines forms quinoid compounds that stimulate the production of superoxide anions and subsequently hydrogen peroxide. In the presence of iron, hydrogen peroxide becomes a highly reactive hydroxyl radical that causes oxidative damage to proteins, lipids, and DNA, thereby impacting the size of the infarcted myocardium [11]. Myocardial necrosis is associated with cardiac dysfunction, increased lipid peroxidation, and altered activities of cardiac enzymes and the antioxidant system [12, 13]. The pathophysiological and morphological aberrations produced in the heart of the myocardial necrotic rat model are comparable with those that occur in human MI [10].

There is an increase in global demand for safe and effective natural products that confer free radical scavenging activities and that offer protection against oxidative stress induced cardiovascular diseases including MI [13]. The dynamic relationship between ROS and antioxidants results from the endogenous antioxidant defense system composed of free radical antioxidant enzymes, such as superoxide dismutase (SOD), catalase, glutathione peroxidase (GPx), glutathione reductase (GRx), and glutathione-S-transferase (GST), which may be lowered due to enhanced lipid peroxidation [8].

In recent years, the prevention of CVD has been linked to the consumption of fresh food items and plants rich in natural antioxidants because of their superior efficacy and safety compared to synthetic products [14]. Honey, a natural liquid produced by honey bees, has received considerable attention recently because it contains at least 181 different substances [15] that are believed to have a therapeutic role in CVD. Its therapeutic value has been partly attributed to its antioxidant properties [16]. The antioxidant constituents of honey include phenolics, flavonoids, ascorbic acid, proteins [17], certain enzymes (glucose oxidase, catalase) [18], *α*-tocopherol, and beta-carotene, a precursor of vitamin A that may reduce the risk of some forms of fatal diseases such as cancer, CVD, and stroke [16].

Tualang honey (TH) is a wild multifloral honey produced by* Apis dorsata* of Asian origin, which form their hives high in tall trees locally known as tualang trees [19]. Previous* in vitro* studies on the antioxidant properties of Malaysian honeys revealed that TH had the highest content of phenolics and flavonoids, as well as the best free radical scavenging properties when compared to other Malaysian honey samples [17, 20]. In addition, the presence of a number of phenolic acids, including gallic, syringic, benzoic, trans-cinnamic, p-coumaric acids, and flavonoid compounds such as catechin and kaempferol, has been identified through high performance liquid chromatography (HPLC) analysis [19], further confirming TH's scientific importance for human health and medicine. While some studies have reported a beneficial role for catechins and kaemferols in cardiovascular function, the cellular mechanisms underlying their biological actions remain unknown [16, 21]. Therefore, the present study was designed to investigate the cardioprotective effects of TH in rats and to identify the possible mechanisms underlying its therapeutic efficacy by studying the alterations in cardiac marker enzymes, troponin I, lipid peroxides, and the antioxidant defense system, in conjunction with histopathological examination.

## 2. Materials and Methods

### 2.1. Experimental Animals

The experiments were conducted using adult male Wistar albino rats (*n* = 40) weighing 160–180 g. The animals were bred and reared in the institutional animal house facility maintained at a constant room temperature of 30 ± 3°C, with humidity ranging between 40% and 70%. The rats were housed in polypropylene cages with hard wood-chip bedding and received a natural 12-h day-night cycle. They were fed a standard pellet diet and water* ad libitum*. The experiments complied with the local institutional ethical guidelines.

### 2.2. Drugs and Chemicals

ISO and 1,1,3,3-tetraethoxy propane were purchased from Nacalai Tesque, Inc., Kyoto, Japan. All chemicals and reagents used in this study were of analytical grade.

### 2.3. Honey Samples

TH samples were provided by the Federal Agricultural Marketing Authority (FAMA) of Malaysia. The dose of honey used in this study was based on previous studies [22, 23].

### 2.4. Experimental Induction of Myocardial Ischemia

Myocardial ischemia was induced by subcutaneous (s.c.) injection of ISO (85 mg/kg dissolved in physiological saline) at an interval of 24 h for two consecutive days. The ISO dose was based on a pilot study for ISO dose fixation as well as results from previous studies [4, 24]. Animals were sacrificed 48 h after the first ISO dose.

### 2.5. Experimental Design

Following acclimatization, the experimental rats were randomly divided into four groups consisting of 10 rats each.

*Group 1 (control)*. Animals received standard laboratory diet and drinking water* ad libitum* and served as a normal control group.

*Group 2 (TH)*. Animals received TH only (3 g/kg) orally for 45 days. (All animals received standard laboratory diet and drinking water* ad libitum*).

*Group 3 (ISO)*. Animals were subcutaneously injected with ISO (85 mg/kg) on the 44th and 45th days (at an interval of 24 h) [24]. (All animals received standard laboratory diet and drinking water* ad libitum*, and this group served as a negative control group).

*Group 4 (TH + ISO)*. Animals were orally treated with TH (3 g/kg) for a period of 45 days followed by subcutaneous injection of ISO (85 mg/kg) on the 44th and 45th days (at an interval of 24 h). (All animals received standard laboratory diet and drinking water* ad libitum*, and this group served as a preventive group).

### 2.6. Serum Preparation

Blood samples (3 mL) were collected in dry test tubes and allowed to coagulate at an ambient temperature for 30 min. Serum was separated by centrifugation at 2000 rpm for 10 min.

### 2.7. Heart Tissue Homogenate Preparation

Immediately following blood collection, the heart samples were separated from the surrounding tissue and were washed twice with ice cold phosphate buffer saline (PBS). The samples were homogenized in phosphate buffer (25 mM, pH 7.4) to produce an approximately 10% w/v homogenate. The homogenate was centrifuged at 1700 rpm for 10 min and the supernatant was collected and stored at −20°C until subsequent biochemical analysis. Some of the heart samples were stored in 10% formalin for histopathological examination.

### 2.8. Serum Biochemical Parameters

The collected serum was used for determination of cardiac troponin I (cTnI) using commercially available enzyme immunoassay kits (JAJ International Inc., USA). The cardiac marker enzymes creatine kinase-MB (CK-MB), aspartate transaminase (AST), and lactate dehydrogenase (LDH) were estimated using commercially available standard assay kits (Standbio Laboratory, Boerne, TX, USA). Serum was also used for the estimation of total cholesterol (TC) and triglycerides (TG) using commercially available standard assay kits (Standbio Laboratory, Boerne, TX, USA).

### 2.9. Biochemical Parameters on Heart Tissue

#### 2.9.1. Oxidative Stress Biomarker (LPO)

Malondialdehyde (MDA) levels were assayed for products of lipid peroxidation (LPO) in heart tissue. MDA, which is referred to as thiobarbituric acid reactive substance (TBARS), was measured at 532 nm according to the method described by Ohkawa et al. [25] and the levels of TBARS were expressed as nmol of MDA per mg of protein. The total protein in heart tissue homogenates was estimated by the Lowry method [26].

#### 2.9.2. Antioxidant Enzymes

The heart tissue homogenate was centrifuged again at 12,000 rpm for 10 min at 4°C using a high speed cooling centrifuge (Eppendorf centrifuge 5415D, Germany). The yielded supernatant was used for estimation of endogenous antiperoxidative enzymes, SOD, GPx, GRx, and GST using standard assay kits (Abnova Corporation, Taiwan). SOD activity was expressed as units/mg of protein, while GPx and GRx activity was expressed as nmol NADPH oxidized/min/mg of protein and GST activity was expressed as nmol CDNB (1-chloro-2,4-dinitrobenzene) conjugated/min/mg of protein.

### 2.10. Histopathological Examination

After the rats were sacrificed, their hearts were rapidly dissected and immediately washed with saline, followed by fixation in 10% formalin. The fixed tissues were embedded in paraffin, and then serial sections of 5 *μ*m thickness were cut. Each section was stained with hematoxylin and eosin (H & E). The microscopic examination was carried out using a normal-spectra fluorescent microscope (Olympus DP72) at 40x. Photomicrographs were then taken with a digital camera (Olympus, Tokyo, Japan) attached to the microscope. To avoid any possible bias, the pathologist performing the histopathological examination was blinded to the treatment assignment of the different study groups.

### 2.11. Statistical Analysis

Data were analyzed using SPSS (Statistical Packages for Social Science, version 20.0, IBM Corporation, New York, USA) and Microsoft Excel 2007 (Redmond, Washington, USA). Statistical analyses of the biochemical data were conducted by using Tukey's test. The results are presented as mean values ± standard deviations (SD). A *p* value of < 0.05 was accepted as statistically significant.

## 3. Results

There were no deaths in any of the experimental groups during the treatment period (45 days).  Table 1 shows that there was no significant difference in body weight (BW) between the different treatment groups; although ISO alone treated rats had a slight reduction in BW gain compared with normal controls, this difference was not statistically significant. ISO administration significantly (*p* < 0.05) increased absolute and relative heart weights compared to normal controls. Pretreatment with TH followed by ISO treatment reduced the absolute and relative heart weights to control levels, although this difference was not statistically significant.

**Figure 1:**
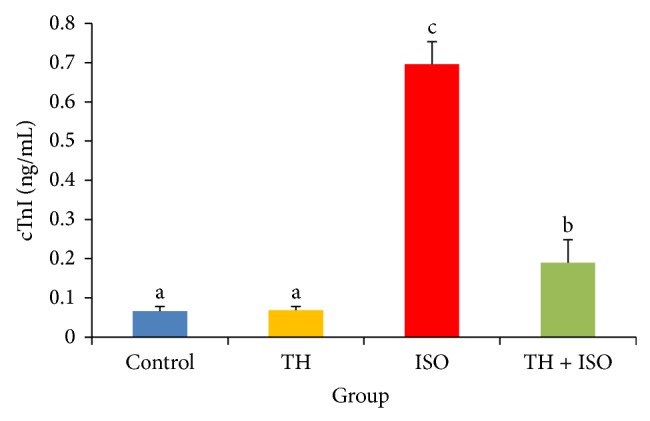
The cardioprotective effects of TH and cTnI levels in rats, measured using ELISA method. The bars represent the mean ± SD (*n* = 10); bars with different letters are significantly different at *p* < 0.05.

Rats injected with ISO alone showed a marked (*p* < 0.05) elevation in the serum cTnI levels when compared to normal controls. However, oral pretreatment of TH to ISO-treated rats for a period of 45 days significantly (*p* < 0.05) decreased serum cTnI levels when compared with ISO and normal control groups (Figure 1).

The induction of MI following ISO administration significantly increased all serum cardiac marker enzyme activities (CK-MB, AST, and LDH) compared to controls. However, oral pretreatment with honey ameliorated all ISO-induced alterations of the diagnostic marker enzymes to normal levels. No significant difference was observed in rats treated with TH alone except for a slight increase in AST activity compared to the control rats (Table 2).

**Figure 2:**
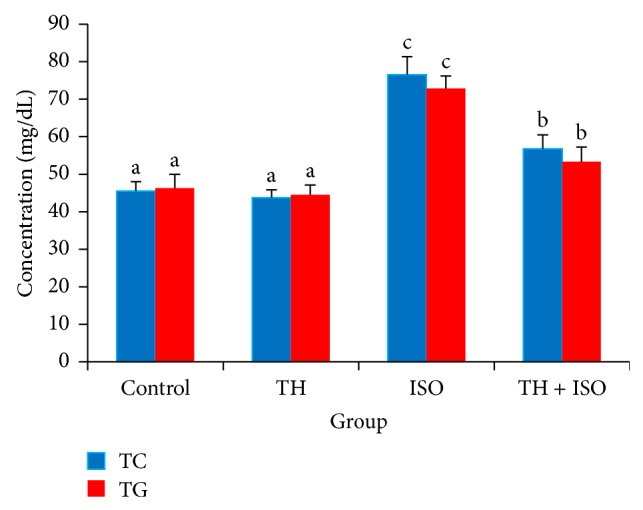
The protective effects of TH on serum TC and TG levels in rats, estimated directly using standard assay kit. The bars represent the mean ± SD (*n* = 10); bars with different letters are significantly different at *p* < 0.05.

Rats treated with ISO alone showed a significant increase in serum TC and TG levels when compared with normal controls. However, honey pretreatment reduced these parameters significantly. In the baseline group, rats treated with honey alone showed slight reductions in serum TC and TG levels when compared to control rats, although this difference was not statistically significant (Figure 2).

ISO significantly increased the levels of MDA compared to normal controls (Table 3). However, a significant diminution of ISO-induced MDA elevation was observed in all honey-pretreated groups. The activities of the antioxidant enzymes SOD, GPx, GRx, and GST in the heart of rats that were only treated with ISO were significantly decreased compared to controls. Pretreatment of ISO-induced rats with honey significantly increased the activities of GPx, GRx, and GST enzymes compared to rats treated with ISO alone.

**Figure 3:**
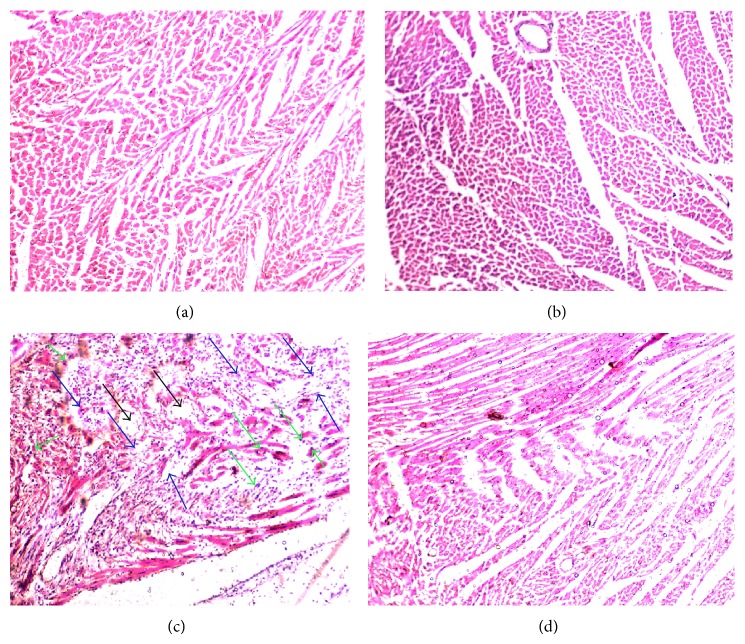
Effect of TH on histopathological changes observed on the ventricular wall of heart muscle. (a) Group 1: photomicrograph of histopathological examination of stained myocardial tissue section from normal control rats showing normal cardiac muscle fibers. (b) Group 2: TH-treated (3 g/kg) heart showing normal cardiac muscle fibers with no pathological changes. (c) Group 3: ISO alone (85 mg/kg) treated heart showing separation of cardiac muscle fibers (blue arrows) and edematous intramuscular spaces (black arrows) with infiltration of inflammatory cells (green arrows). (d) Group 4: honey + ISO-treated heart, indicating that TH conferred some cardioprotection; markedly increased areas of normal muscle fibers can be observed. (Magnification: 40x).

Figures 3(a)–3(d) show the effects of TH on the histopathology of heart tissue in normal and ISO-induced MI rats. Normal cardiac muscle fibers with cellular integrity were observed in normal untreated rats (Figure 3(a)). Normal rats treated with honey alone also showed normal cardiac muscle bundles, with no evidence of infarction or tissue damage (Figure 3(b)). Histopathological evaluation confirmed induction of MI by ISO with the area of infarction containing split cardiac muscle fibers, edematous intramuscular spaces, and inflammatory infiltrates (Figure 3(c)). Pretreatment with honey (85 mg/kg) for 45 days, however, decreased the degree of infiltration of inflammatory cells, and the treated rats showed relatively well-preserved cardiac muscle fiber morphology (Figure 3(d)).

## 4. Discussion

To our knowledge, our study is the first to demonstrate that TH has cardioprotective effects. Investigation of the hearts and their relative organ weights revealed a significant increase in both absolute and relative weights following ISO administration, although the body weight remained relatively unchanged. The increase in heart weight may be attributed to increased water accumulation in edematous intramuscular spaces in cardiac tissue and increased protein content [27], which was also confirmed on histopathological examination. These results are consistent with a previous report on ISO-induced myocardial ischemia [13]. It has been proposed that myocardial function may be reduced by approximately 10% with a 1% increase in myocardial water content [28]. Pretreatment with TH, however, slightly decreased the relative heart weight to near normal, which is indicative of its protective effect on the myocardium.

It has been reported that ISO-induced cytotoxic free radical generation and the development of infarct-like lesions of the subendocardium with coronary insufficiency have been observed in experimental animal studies [10, 13]. Flavonoids such as catechin and kaempferol, phenolic acids, ascorbic acid, and proteins are important constitutive antioxidants that have been detected in TH [17, 19], all of which can work synergistically to scavenge and eliminate free radicals [29]. It is plausible that the presence of these antioxidants may help to protect against oxidative cardiac injury, thus restricting the leakage of these enzymes from the myocardium.

cTnI is a low molecular weight protein constituent of the myofibrillary contractile apparatus of the cardiac muscle. It is a sensitive and a highly specific diagnostic marker of MI [30, 31]. Elevated cTnI levels can predict the risk of both cardiac death and subsequent infarction and can serve as a predictor of myocardial ischemia in patients subjected to a stress test [8, 32]. In our study, increased cTnI levels were observed in the serum of ISO-induced rats. Our results are consistent with a previous study reported by Priscilla and Prince [8]. Pretreatment with honey significantly decreased serum cTnI levels in ISO-induced cardiotoxic rats. This result may be due to the protective effect of TH on the myocardium through preservation of the structural and functional integrity of the contractile apparatus, thereby preventing cardiac injury and leakage of troponins from myocardium into the blood circulation. Further investigation of the mechanisms underlying the cardioprotective effects of TH is warranted.

The myocardium contains ample concentrations of diagnostic markers of MI and, when damaged, releases its contents into the extracellular fluid [27]. Once myocardial cells are damaged or destroyed due to insufficient oxygen supply or nutrients, the cardiac membrane becomes permeable or may rupture, resulting in leakage of cytosolic enzymes into the blood stream with concomitant increases of their serum concentrations [33]. Consistent with previous studies [13, 31], in the present study, ISO administration to rats caused marked elevations in all of the serum cardiac marker enzyme activities. CK-MB activity in the serum is a sensitive and important diagnostic marker of MI because of its abundance in myocardial tissue and its virtual absence from most other tissues [34]. Pretreatment with honey significantly lowered the ISO-induced elevation of serum levels of the cardiac marker enzymes, demonstrating that TH maintains membrane integrity and restricts the leakage of these cardiac enzymes. No significant changes were observed in cardiac markers in rats treated solely with TH. Although serum AST activity was slightly elevated, other diagnostic parameters strongly supported the ameliorative effect of TH honey on oxidative stress.

An important factor in CVD is the key role that lipids play in the pathogenesis of CVD not only via hyperlipidemia and the development of atherosclerosis but also by modifying the composition, structure, and stability of cellular membranes [35]. ISO-induced rats exhibited significant elevation in serum total cholesterol and triglycerides. These alterations could be attributed to enhanced biogenesis of lipids by the cardiac cAMP cascade [36]. Our observations are consistent with previous reports by Patel et al. [13] and Radhiga et al. [4]. Honey pretreatment restored TC and TG levels in treatment rats. Alagwu et al. [37] speculated that honey increases bile cholesterol excretion and lowers plasma cholesterol levels, though this observation requires further investigation.

ISO auto-oxidation leads to the generation of excessive amounts of ROS that may primarily target and attack PUFAs within the cellular membranes and form peroxy radicals that in turn attack adjacent phospholipids in a chain reaction of lipid peroxidation [8]. Lipid peroxidation is an important pathogenic event in MI [38]. MDA is a major lipid peroxidation end product. Increased MDA concentrations may result from increased levels of free radicals and/or decreased activities of the antioxidant defense system [39]. In line with previous reports [8, 13, 31], in the present study, ISO administration caused a marked elevation in LPO, which was expressed as MDA content. Oral pretreatment of rats with honey led to a significant reduction in myocardial MDA content. This result can be attributed to the free radical scavenging activity of TH, by the synergistic scavenging effects of natural honey maybe due to both enzymatic (e.g., catalase, glucose oxidase, and peroxidase) and nonenzymatic (e.g., phenolic acids, flavonoids, ascorbic acid, carotenoids) antioxidants involved in cardiovascular defense mechanisms [40, 41].

Regarding the levels of antiperoxidative enzymes, SOD activity or glutathione redox system which consists of both GPx and GRx [42], it is plausible that the observed decreased activities of SOD, GPx, GRx, and GST in the myocardium of ISO-treated rats may be due to increased generation of ROS, such as superoxide and H_2_O_2_. This in turn leads to inhibition of these enzymes and results in decreased removal of superoxide radicals, H_2_O_2_ radicals, and highly potent hydroxyl radicals [43]. Honey pretreatment restored the decreased levels of these enzymes in heart tissue suggesting that TH provides protection from deleterious effects induced by free radicals in ISO-treated rats. This result could be due to the direct free radical scavenging potency of honey [41]. The possible mechanisms through which honey supplementation restores antioxidant enzyme function may be due to upregulation of the activity or expression of Nrf2 [44], a transcription factor that is released from its repressor (Keap1) under oxidative or xenobiotic stress [45]. The released Nrf2 binds to the antioxidant response element (ARE) of cytoprotective genes and induces their expression as well as subsequent induction of free radical scavenging enzymes to neutralize and eliminate the cytotoxic oxidants [45, 46].

Histopathological examination of myocardial tissue in ISO-treated rats revealed coagulative necrosis of myocytes and separation of cardiac muscle fibers with inflammatory cell infiltration. In comparison, the nearly normal cardiac muscle fiber architecture with reduced inflammatory infiltrates further confirmed the cardioprotective effect of TH. Similar histopathological findings were observed in ISO-treated rats for gallic acid [8], which also has high antioxidant properties. Overall, the results of this study offer scientific evidence of the importance of TH in cardioprotection against CVD, a disease whose pathogenesis has long been associated with oxidative stress.

## 5. Conclusion

The present study demonstrated the protective effect of TH in ISO-induced MI in rats, as evidenced by its protective effect on the release of cardiac markers in serum. In addition, our study provided experimental evidence that TH improved the antioxidant enzyme levels in heart tissue and lowered LPO levels following exposure to high dose ISO. These biochemical findings were further confirmed by histopathological examination of both Tualang-honey-pretreated and ISO-treated hearts. The present study demonstrated that the cardioprotective effect of Tualang honey against ISO-induced oxidative stress could be contributed by the presence of endogenous antioxidant enzymes via inhibition of lipid peroxidation.

## Figures and Tables

**Table 1:** The effects of TH on BW, absolute, and relative heart weights.

Parameters	Treatment			
	Control	TH	ISO	TH + ISO
Initial BW(g)	169.00 ± 7.75^a^	170.40 ± 6.89^a^	171.00 ± 4.85^a^	169.90 ± 8.11^a^
Final BW (g)	239.50 ± 8.46^a^	237.10 ± 8.24^a^	236.20 ± 7.17^a^	237.00 ± 4.59^a^
BW gain (%)	29.30 ± 4.29^a^	27.50 ± 3.09^a^	27.50 ± 3.26^a^	28.28 ± 3.91^a^
Absolute heart weight (g)	0.99 ± 0.07^a^	0.97 ± 0.08^a^	1.22 ± 0.01^b^	1.11 ± 0.14^b^
Relative heart weight (g/100 g)	0.42 ± 0.02^a^	0.41 ± 0.02^a^	0.52 ± 0.01^b^	0.48 ± 0.05^b^

Results were expressed as the mean ± SD, *n* = 10. ^a,b,c^Values in the same row that do not share superscript letters (a, b, and c) differ significantly at *p* < 0.05; % of body weight (BW) gain = [(final BW − initial BW)/final BW] × 100. TH: Tualang Honey; ISO: isoproterenol.

**Table 2:** The protective effects of TH on serum levels of cardiac marker enzymes in rats.

Parameters	Treatment			
	Control	TH	ISO	TH + ISO
CK-MB (U/L)	94.44 ± 3.05^a^	90.31 ± 6.15^a^	242.39 ± 7.30^c^	158.03 ± 7.59^b^
AST (U/L)	23.13 ± 2.55^a^	29.87 ± 1.36^b^	110.08 ± 4.95^d^	43.62 ± 3.54^c^
LDH (U/L)	79.82 ± 3.46^a^	81.98 ± 3.95^a^	127.44 ± 3.63^c^	96.20 ± 5.40^b^

The results are expressed as the mean ± SD, *n* = 10.

^a,b,c,d^Values in the same row that do not share superscript letters (a, b, c, and d) differ significantly at *p* < 0.05; TH: Tualang honey; ISO: isoproterenol.

**Table 3:** The protective effects of TH on lipid peroxide (LPO) levels and activities of superoxide dismutase (SOD), glutathione reductase (GRx), glutathione peroxidase (GPx), and glutathione-S-transferase (GST) in the hearts of normal and treated rats.

Parameters	Treatment			
	Control	TH	ISO	TH + ISO
LPO (nmol TBARS/mg of protein)	9.25 ± 0.68^a^	9.33 ± 0.96^a^	22.42 ± 1.09^c^	16.84 ± 1.06^b^
SOD (units/mg of protein)	1.46 ± 0.05^a^	1.49 ± 0.09^a^	0.16 ± 0.01^b^	0.19 ± 0.02^b^
GPx (nmol NADPH oxidized/min/mg of protein)	2.86 ± 0.06^c^	2.83 ± 0.05^c^	1.07 ± 0.19^a^	1.86 ± 0.20^b^
GRx (nmol NADPH oxidized/min/mg of protein)	83.66 ± 2.73^b^	85.33 ± 2.18^bc^	76.59 ± 5.92^a^	89.05 ± 5.01^c^
GST (nmol CDNB conjugated/min/mg of protein)	2.39 ± 0.21^b^	3.58 ± 0.04^a^	0.85 ± 0.02^c^	1.89 ± 0.23^b^

Results are expressed as the mean ± SD, *n* = 10.

^a,b,c^Values in the same row that do not share superscript letters (a, b, and c) differ significantly at *p* < 0.05; TH: Tualang honey; ISO: isoproterenol.
