# Expression of Concern on “Cardioprotective Effects of Tualang Honey: Amelioration of Cholesterol and Cardiac Enzymes Levels”

**DOI:** 10.1155/bmri/9857817

**Published:** 2026-08-14

**Authors:** BioMed Research International

EXPRESSION OF CONCERN: M. I. Khalil, E. M. Tanvir, R. Afroz, S. A. Sulaiman, S. H. Gan, “Cardioprotective Effects of Tualang Honey: Amelioration of Cholesterol and Cardiac Enzymes Levels,” *BioMed Research International*, 2015, 286051, https://doi.org/10.1155/2015/286051

This Expression of Concern is for the above article, published online on 03 May 2015 in Wiley Online Library (https://onlinelibrary.wiley.com/), and has been issued by agreement between the journal Editor‐in‐Chief, Yoel Klug; and John Wiley & Sons Ltd. The Expression of Concern has been agreed due to multiple concerns identified in Figure 3, some of which are raised on PubPeer [1]. Further investigation identified additional concerns with the Figure, specifically, Panel C appears identical to panel D in Figure 5 of [2], and panel C in Figure 7 of [3].

Following assessment of the concerns raised, the authors’ response, and the absence of original image data, the publisher was unable to resolve concerns regarding the apparent reuse of image panels across multiple publications from the same research group.

As these concerns could not be satisfactorily addressed, the publisher has issued this Expression of Concern to inform readers.

## References

[1] Arachidis M. , Cardioprotective Effects of Tualang Honey: Amelioration of Cholesterol and Cardiac Enzymes Levels, PubPeer. (2023) https://pubpeer.com/publications/83477C1259E760550C96CD7377D0C4. 10.1155/2015/286051 PMC4433628 26064893

[2] Afroz R. , Tanvir E. M. , Karim N. , Hossain M. S. , Alam N. , Gan S. H. , and Khalil M. I. , Sundarban Honey Confers Protection against Isoproterenol-Induced Myocardial Infarction in Wistar Rats, BioMed Research International. (2016) 6437641, 10.1155/2016/6437641. PMC4886051 27294126

[3] Ahmed R. , Tanvir E. M. , Hossen M. S. , Afroz R. , Ahmmed I. , Rumpa N.-E.-N. , Paul S. , Gan S. H. , Sulaiman S. A. , and Khalil M. I. , Antioxidant Properties and Cardioprotective Mechanism of Malaysian Propolis in Rats, Evidence-Based Complementary and Alternative Medicine. (2017) 5370545, 10.1155/2017/5370545. PMC5312504 28261310

